# Deep learning-assisted diagnosis of benign and malignant parotid gland tumors based on automatic segmentation of ultrasound images: a multicenter retrospective study

**DOI:** 10.3389/fonc.2024.1417330

**Published:** 2024-08-09

**Authors:** Wei Wei, Jingya Xu, Fei Xia, Jun Liu, Zekai Zhang, Jing Wu, Tianjun Wei, Huijun Feng, Qiang Ma, Feng Jiang, Xiangming Zhu, Xia Zhang

**Affiliations:** ^1^ Department of Ultrasound, The First Affiliated Hospital of Wannan Medical College (Yijishan Hospital), Wuhu, China; ^2^ Department of Radiology, The First Affiliated Hospital of Wannan Medical College (Yijishan Hospital), Wuhu, China; ^3^ Department of Ultrasound, WuHu Hospital, East China Normal University (The Second People’s Hospital, WuHu), Wuhu, Anhui, China; ^4^ Department of Ultrasound, Linyi Central Hospital, Linyi, Shandong, China; ^5^ Department of Ultrasound, Zibo Central Hospital, Zibo, Shandong, China

**Keywords:** automatic segmentation, deep learning, parotid gland tumors, ultrasound, net reclassification index, integrated discrimination improvement

## Abstract

**Objectives:**

To construct deep learning-assisted diagnosis models based on automatic segmentation of ultrasound images to facilitate radiologists in differentiating benign and malignant parotid tumors.

**Methods:**

A total of 582 patients histopathologically diagnosed with PGTs were retrospectively recruited from 4 centers, and their data were collected for analysis. The radiomics features of six deep learning models (ResNet18, Inception_v3 etc) were analyzed based on the ultrasound images that were obtained under the best automatic segmentation model (Deeplabv3, UNet++, and UNet). The performance of three physicians was compared when the optimal model was used and not. The Net Reclassification Index (NRI) and Integrated Discrimination Improvement (IDI) were utilized to evaluate the clinical benefit of the optimal model.

**Results:**

The Deeplabv3 model performed optimally in terms of automatic segmentation. The ResNet18 deep learning model had the best prediction performance, with an area under the receiver-operating characteristic curve of 0.808 (0.694−0.923), 0.809 (0.712−0.906), and 0.812 (0.680−0.944) in the internal test set and external test sets 1 and 2, respectively. Meanwhile, the optimal model-assisted clinical and overall benefits were markedly enhanced for two out of three radiologists (in internal validation set, NRI: 0.259 and 0.213 [*p* = 0.002 and 0.017], IDI: 0.284 and 0.201 [*p* = 0.005 and 0.043], respectively; in external test set 1, NRI: 0.183 and 0.161 [*p* = 0.019 and 0.008], IDI: 0.205 and 0.184 [*p* = 0.031 and 0.045], respectively; in external test set 2, NRI: 0.297 and 0.297 [*p* = 0.038 and 0.047], IDI: 0.332 and 0.294 [*p* = 0.031 and 0.041], respectively).

**Conclusions:**

The deep learning model constructed for automatic segmentation of ultrasound images can improve the diagnostic performance of radiologists for PGTs.

## Introduction

Salivary gland tumors (SGTs) are relatively uncommon diseases representing 3-12% of head and neck tumors (1), which most frequently occur in the parotid gland, an important exocrine organ. As data show, parotid gland tumors (PGTs) are the most common type of SGTs, which are approximately 80% benign and 20% malignant (2). The most common malignant PGTs (MPTs) are mucoepidermoid carcinoma and adenoid cystadenocarcinoma, while the most common benign PGTs (BPTs) are pleomorphic adenoma and Warthin tumor (3). Currently, the treatment for PGTs is predominantly surgical, and their treatment strategies and prognosis vary by histopathological types (4, 5). Specifically, BPTs are treated with local excision or lateral parotidectomy, while MPTs require more radical surgery such as extended resection and lymph node dissection. Therefore, accurate preoperative diagnosis of BPTs and MPTs is of utmost importance in adjusting treatment decisions.

As all PGTs are generally asymptomatic, their nature is distinguished before surgery primarily by ultrasound-guided core needle biopsy (USCB) or fine-needle aspiration (FNA) and medical imaging (6–9). Although USCB and FNA are considered minimally invasive and safe, they confer a risk of facial nerve injury, mumps, and tumor cell spread along the needle track, with the disadvantage of limited sampling that causes uncertain histological or cytological diagnosis (7, 8). Currently, preoperative imaging for PGTs includes ultrasound, magnetic resonance imaging (MRI), and computed tomography (CT). Despite the higher image quality of CT and MRI in differentiating parotid gland lesions (10), they also have some limitations, including exposure to ionizing radiation, contraindications for patients with internal ferromagnetic devices, high monetary cost, and prolonged examination times. These limitations have restricted the clinical use of CT and MRI in evaluating therapeutic effects in patients with PGTs (11, 12).

Notably, ultrasound is ideally applicable to patients with PGTs since PGTs are typically located in the superficial lobe and ultrasound is widely accepted to be preferred for superficial organ examination. Additionally, ultrasound has the advantages of low cost, non-invasiveness, and no ionizing radiation (12). Therefore, ultrasound is often used for the diagnosis of PGTs. Nevertheless, differential diagnosis of PGTs by ultrasound is challenging. A prior study exhibited that the accuracy of ultrasound in diagnosing malignant parotid masses was only 20%, despite its sensitivity and specificity of 38.9% and 90.1%, respectively, in diagnosing parotid masses (13). Therefore, it is urgent to develop a more reliable, non-invasive, and rapid method for determining the nature of PGTs.

Recently, machine learning (ML) has been increasingly used to analyze medical images. Deep learning (DL), an essential technology in ML, involves multiple levels, which has attracted wide attention because it can automatically learn semantic and spatial features of the hidden layers of neural networks (14). Intriguingly, several studies have yielded similar results in terms of the diagnostic performance of DL systems in the medical imaging diagnosis of various diseases, including thyroid diseases (15), breast diseases (16), and liver tumors (17). Additionally, some previous studies have revealed that DL can discriminate PGTs using medical image pairs, mainly involving CT (6, 18) and MRI (1). Yet, few studies have assessed the performance of DL methods based on ultrasound images in distinguishing BPTs from MPTs. Basically, images of PGTs obtained by a method involving DL are manually segmented by radiologists, which has two chief drawbacks. First, manual segmentation is quite time-consuming and error-prone. Second, accurate segmentation heavily depends on the subjectivity of radiologists, signifying that image segmentation quality is physician-dependent (19). Accordingly, it is imperative to develop a model integrating the optimal automatic segmentation model and DL methods for improving workflow efficiency and assisting radiologists in diagnosis. This study determined regions of interest (ROI) with an automatic segmentation algorithm, evaluated popular image segmentation algorithms including Deeplabv3, UNet, and UNet++, and constructed a deep learning-assisted diagnostic model for PGTs based on automatic segmentation of ultrasound images.

## Materials and methods

### Patients

**Figure 1:**
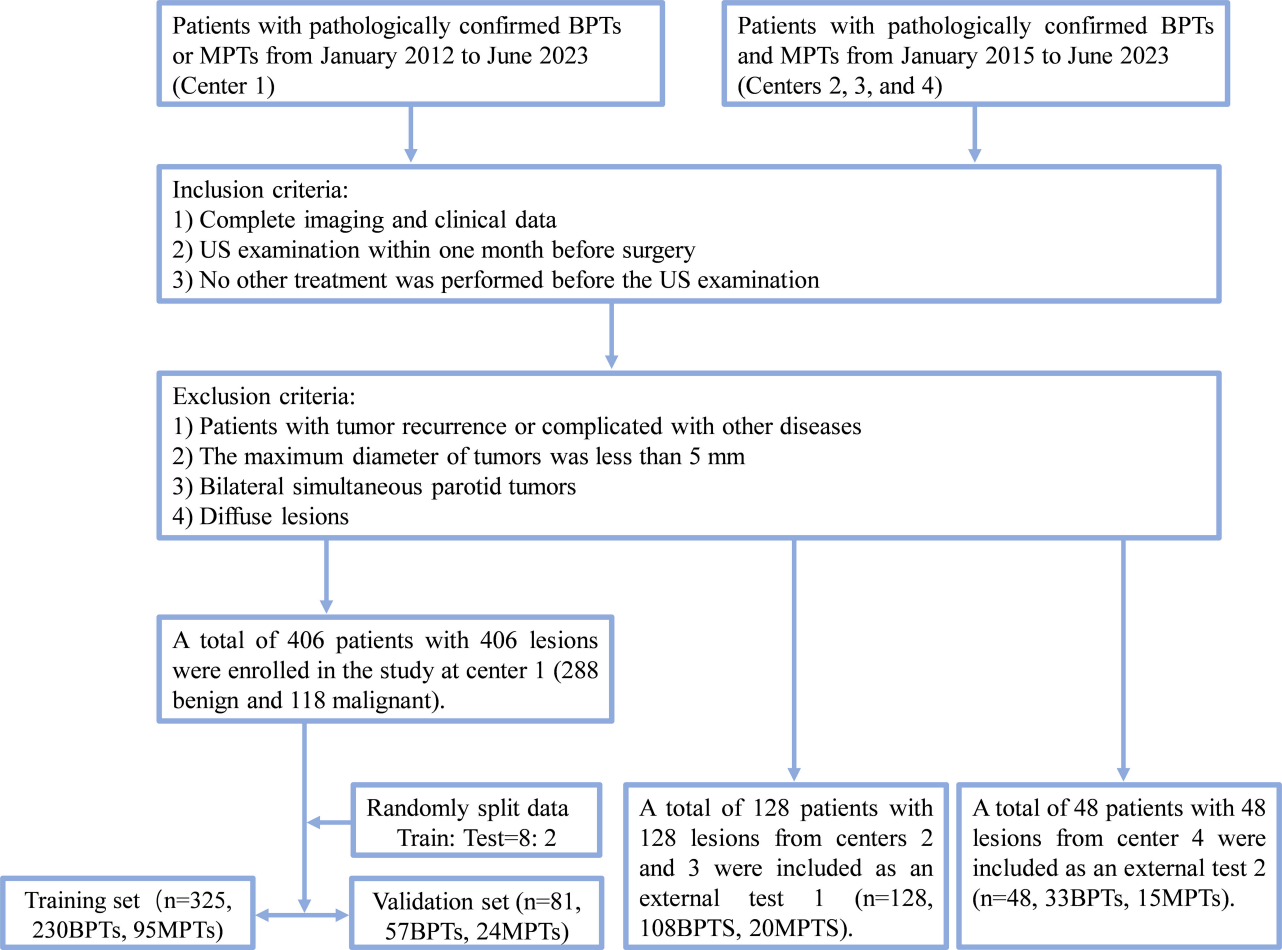
Flow chart of patient recruitment. Center 1, the First Affiliated Hospital of Wannan Medical College (Yijishan Hospital); External test set 1: center 2, WuHu Hospital, East China Normal University (The Second People’s Hospital, WuHu) and center 3, the Zibo Central Hospital; External test set 2, the Linyi Central Hospital. BPTs, benign parotid gland tumors; MPTs, malignant parotid gland tumor.

A total of 582 patients histopathologically diagnosed with BPTs and MPTs were retrospectively recruited from four centers (**Figure 1**), consisting of 406 patients from center 1, 95 patients from center 2, 33 patients from center 3 (patients in centers 2 and 3 datasets were included in external test set 1), and 48 patients from center 4 (**Supplementary Table S1** for classification of histopathological types and numbers). Inclusion criteria for patients were as follows (1): patients with complete imaging and clinical data; (2) patients who received ultrasound within one month before surgery; (3) patients who did not receive any other treatments before the ultrasound. Exclusion criteria for patients were as follows: (1) patients with tumor recurrence or complicated with other diseases; (2) patients with tumors less than 5 mm in maximum diameter; (3) patients with simultaneous bilateral PGTs; (4) patients with diffuse lesions.

### Ultrasonic instrument

Ultrasound of all patients was performed on ultrasound equipment (**Supplementary Table S2**) at these 4 centers.

### Ultrasound image acquisition

All ultrasound images were stored in JPG or BMP format in the picture archiving and communication system (PACS). Before ultrasound, patients were placed in a supine position, with the neck slightly tilted to fully expose the parotid gland examination area. The sonographer performed crossing, multi-section, and bilateral contrast scanning of the parotid gland. The following features of the lesion were visualized with conventional ultrasound: maximum diameter, side (left/right), number (single/multiple), margin (clear/unclear), shape (regular/irregular), echo (uniform/uneven), posterior echogenicity enhancement (presence/absence), cystic or necrotic areas (presence/absence), calcification (presence/absence), Alder (20) grade (Alder 0/I/II/III), abnormal lymph nodes (presence/absence).

### Clinical model construction and validation

Statistical analysis was conducted. The independent samples *t*-test was used only for comparisons of continuous variables between two groups, and the χ² test was utilized to analyze discrete variables. All clinical characteristics were subjected to univariate analyses, followed by the calculation of odds ratios (OR) and corresponding *p* values for each variable. Then, clinical characteristics with *p* < 0.05 were included to construct clinical models. The features identified in the univariate analyses were modeled with three ML algorithms (Logistic Regression [LR], Random Forest, and XGBoost).

### Analysis of radiological and clinical data

The images and clinical data of all patients were attained from the routine clinical records and PACS of hospitals. All images were stored. Combined with the medical history of patients, the lesions were directly evaluated by three physicians from center 1 (radiologist A: XZ, a senior radiologist with 20 years of ultrasound experience; radiologist B: FHJ, an attending radiologist with 13 years of ultrasound experience; radiologist C: JW, a junior radiologist with 5 years of ultrasound experience). Meanwhile, all sonographic features were independently reviewed by ultrasound radiologists A and B, and all images were read by two radiologists in a double-blind manner. If the two radiologists disagreed on the image features of the lesion, they consulted and reached a consensus. Afterward, DL analysis was conducted with B-mode images containing lesions with the largest diameter or planes of suspected malignant features. Radiologists were blinded to the final histopathological findings throughout the study.

### ROI segmentation

ROIs were identified with an automatic segmentation algorithm. Common image segmentation algorithms, including Deeplabv3, UNet, and UNet++, were evaluated. Additionally, different algorithms were combined with a post-fusion algorithm to obtain more accurate ROI segmentation results.

### Training process

Data augmentation was performed. Concretely, sub-volumes were randomly cropped from the images and labeled according to their positive and negative labels, with spatial size and number of samples specified. Online data augmentation was used during the training process, such as spacing and random crop methods, which generated many different images as training iterations. DiceCELoss combining Dice Loss and Cross-Entropy Loss functions (Loss functions in **Supplementary S3**) was utilized. The weights of unlabeled pixels were set to zero, enabling unlabeled pixels to learn from only the labeled ones and, hence, to be generalized to the whole volume.

Hyperparameters were selected. The Adam optimizer was used at an initial learning rate of 1e-3. Our model was trained with 32 rounds of early stopping.

### DL procedure

**Figure 2:**
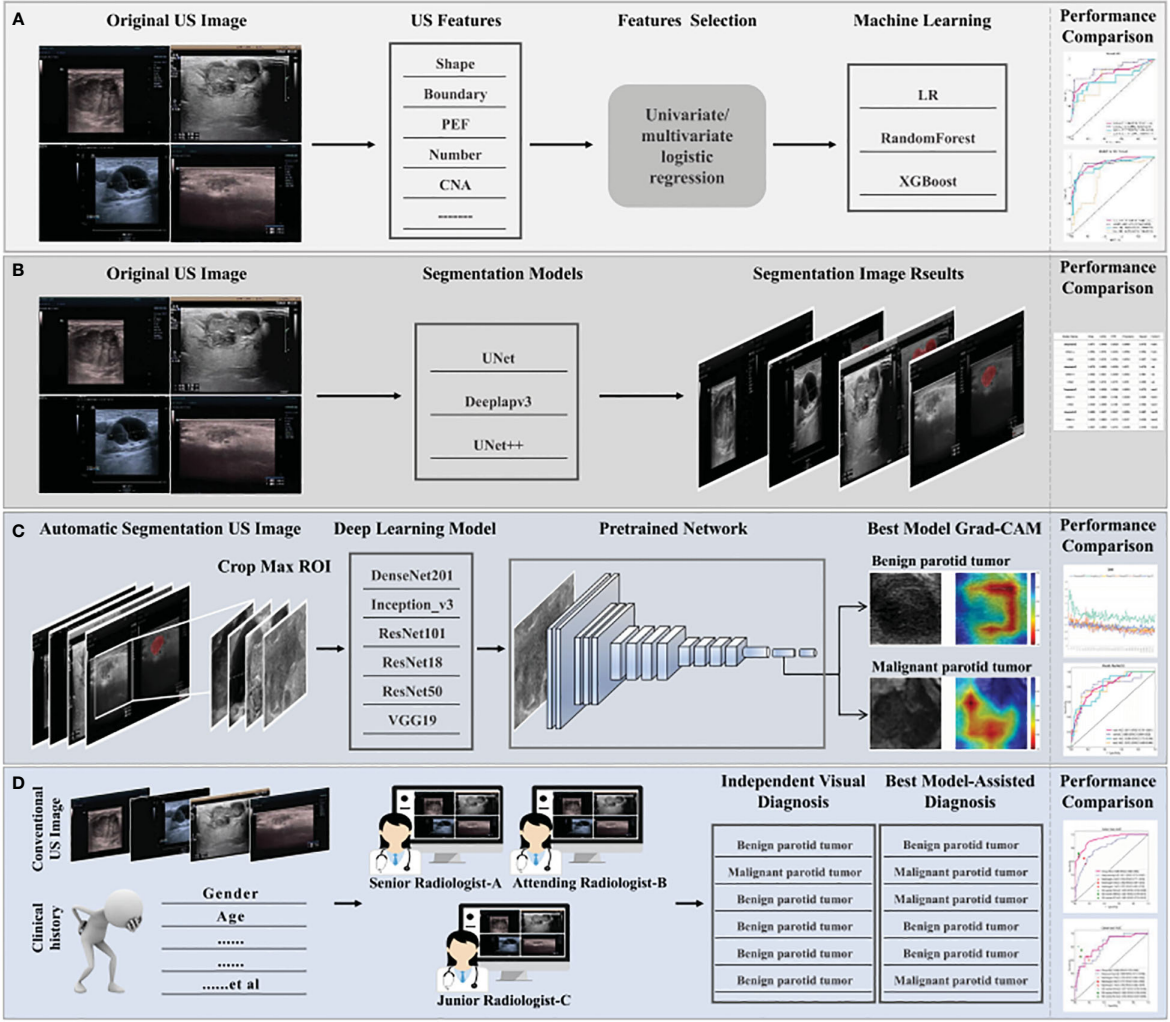
The overall flow of this study. **(A)** Construction process for clinical machine learning model of baseline ultrasound features. **(B)** Selection of segmentation models, automatic segmentation algorithms including Deeplabv3, UNet, and UNet++, to identify regions of interest. Optimal segmentation modeling results were obtained with quantization. **(C)** The construction process for the deep learning model. Ultrasound images were obtained with the results based on the automatic segmentation model and used as the input of different pre-trained models, six classical convolutional neural networks (DenseNet 121, Inception_V3, ResNet 101, ResNet18, ResNet 50, and VGG19). The predicted probability for BPTs and MPTs was the output. In addition, Grad-CAM (gradient-weighted class activation mapping) was applied to visualize the decision-making process of the model. **(D)** Three radiologists with different years of experience provided a comprehensive diagnosis of MPTs and BPTs, with or without the aid of the model. Radiologist C represents resident radiologists. Radiologist B represents attending radiologists. Radiologist A represents senior radiologists.

All 2D rectangular ROIs were cropped from raw US images as per the 2D automatic segmentation mask of the tumor. **Figure 2** summarizes the overall flow of this study. Five commonly used pre-trained convolutional neural network (CNN) models including DensNet201, Inception_V3, ResNet101, ResNet18, ResNet50, and VGG19 were used and all initially trained in the ILSVRC-2012 dataset. The slice with the largest ROI was chosen to represent each patient. After that, gray values were normalized to the range [-1, 1] through min-max transformation. Each cropped sub-volume image was resized to 224 × 224 with nearest-neighbor interpolation to obtain images suited for model input. Due to the limited image data, the learning rate was determined with caution to improve generalization. In this study, the cosine decay learning rate algorithm was implemented (learning rate in **Supplementary S4**).

To increase the visibility of DL models and deepen the understanding of the underlying decision-making process, the goal of achieving model visualization and verification is of tremendous significance in model development (21). Therefore, to more transparently study the interpretability of DL-based radiomics, the model was visualized with gradient-weighted class activation mapping (Grad-CAM). The gradient information from the last convolutional layer of CNN was used for weighted fusion to acquire a class activation map, highlighting the important regional images for the classification target.

The entire training process was performed on an NVIDIA 4090 GPU with the use of MONAI 0.8.1 and PyTorch 1.8.1.

### Visual evaluations and DL model–assisted diagnosis of radiologists

All ultrasound images of PGTs were reviewed independently by three radiologists (radiologists A, B, and C) who were blinded to the histopathological findings. These radiologists comprehensively interpreted the condition of the recruited patients with BPTs or MPTs based on ultrasound images and clinical information, combined with or without the prediction of the optimal model. In the process of our test, our overall process is as follows: step 1: provides two main types of information. First of all, the ultrasound images and clinical data. Secondly, the prediction results of the AI model are presented, focusing on the prediction label (probability of benign or malignant; for example, 0.147 for BPT and 0.853 for MPT) and the corresponding confidence score. step 2: At the request of the radiologist, we provided 2 pieces of supplementary information. The first is the Grad-CAM visualization generated by our deep learning model, which helps radiologists initially focus on salient parts of the image. The second is to provide by our automatic segmentation algorithm to identify region of interest (ROI). Additionally, under the assistance of the optimal DL model, the independent diagnosis was carried out again in a random order. After the assistance of the model, the values of Net Reclassification Index (NRI) and Integrated Discrimination Improvement (IDI) were calculated for the three radiologists to assess the clinical benefit.

### Statistical analysis

The data were analyzed with Python software (version 3.9.16), R software (4.1.0), and SPSS software (version 27.0, IBM Corporation, Armonk, New York, USA). Differences were considered statistically significant at two-sided *p* < 0.05. Statistical analysis is detailed in the **Supplementary S5**.

## Results

### Analysis of baseline characteristics and the XGBoost model

**Figure 3:**
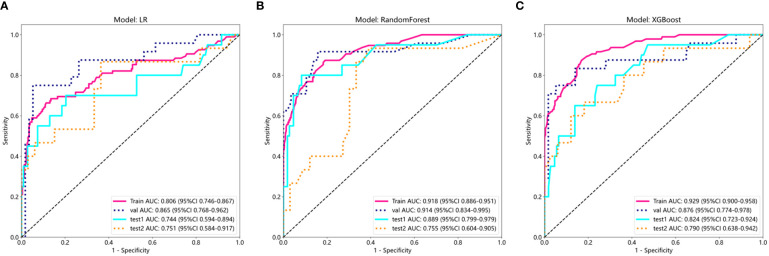
ROC results of clinical features of three different machine learning models (LR, Random Forest, and XGBoost). **(A)** ROC curve of logistic regression (LR); **(B)** ROC curve of Random Forest; **(C)** ROC curve of XGBoost.

**Supplementary Table S3** shows Cohen’s kappa consistency analysis results of subjective morphological features of ultrasound by senior radiologists and attending radiologists (radiologists A and B). The baseline clinical radiological characteristics are listed in **Table 1**. Univariate analyses were performed on all clinical features, and OR and corresponding *p* values were calculated for each variable. Features including shape, echogenicity, calcification, margin, and lymphatic metastasis (LM) had *p*-values below 0.05. Then, multivariate logistic regression analyses were conducted to screen the independent risk factors for predicting MPTs, followed by the construction of a clinical diagnostic model. It was found that shape, margin, and LM were independent risk factors for ultrasound diagnosis of MPTs (**Supplementary Table S4**). Therefore, clinical models were constructed based on these features with three ML algorithms. The results manifested that the performance of the nonlinear model was superior to that of the linear model. The XGBoost model was chosen as the benchmark for clinical characteristics in subsequent comparisons (**Table 2**, **Figure 3**).

**Table 1:** Baseline clinical-radiological characteristics of these data sets.

clinical -radiological feature	Training set (n = 325)		*p*-value	Internal validation set (n = 81)		*p*-value	External test set 1 (n = 128)		*p*-value	External test set 2 (n = 48)		*p*-value
	BPTs (n = 230)	MPTs (n= 95)		BPTs (n = 57)	MPTs (n = 24)		BPTs (n = 108)	MPTs (n = 20)		BPTs (n = 33)	MPTs (n = 15)	
Age	49.47 ± 15.49	52.78 ± 15.75	0.105	52.79 ± 15.56	61.46±14.50	0.0216	53.48 ± 13.56	56.55 ± 10.55	0.491	54.97 ± 12.18	61.80 ± 13.70	0.097
Size	25.69 ± 10.23	26.64 ± 10.65	0.413	27.77 ± 8.85	27.54±12.47	0.541	27.85 ± 11.57	23.02 ± 7.59	0.164	29.70 ± 11.92	34.47 ± 12.88	0.216
Gender			0.933			0.538			0.0178			0.932
0	107 (46.52)	43 (45.26)		18 (31.58)	10(41.67)		32 (29.63)	12 (60.00)		9 (27.27)	5 (33.33)	
1	123 (53.48)	52 (54.74)		39 (68.42)	14(58.33)		76 (70.37)	8 (40.00)		24 (72.73)	10 (66.67)	
Side			0.363			0.617			0.224			0.720
0	174 (75.65)	77 (81.05)		45 (78.95)	17(70.83)		51 (47.22)	13 (65.00)		21 (63.64)	8 (53.33)	
1	56 (24.35)	18 (18.95)		12 (21.05)	7(29.17)		57 (52.78)	7 (35.00)		12 (36.36)	7 (46.67)	
Shape			< 0.001			<0.001			< 0.001			0.002
0	143 (62.17)	24 (25.26)		40 (70.18)	3(12.50)		85 (78.70)	7 (35.00)		24 (72.73)	3 (20.00)	
1	87 (37.83)	71 (74.74)		17 (29.82)	21(87.50)		23 (21.30)	13 (65.00)		9 (27.27)	12 (80.00)	
Number			0.651			0.590			0.525			0.292
0	213 (92.61)	90 (94.74)		48 (84.21)	22(91.67)		101 (93.52)	20 (100.00)		28 (84.85)	10 (66.67)	
1	17 (7.39)	5 (5.26)		9 (15.79)	2(8.33)		7 (6.48)	null		5 (15.15)	5 (33.33)	
Margin			< 0.001			< 0.001			< 0.001			0.0480
0	192 (83.48)	33 (34.74)		52 (91.23)	6(25.00)		98 (90.74)	9 (45.00)		28 (84.85)	8 (53.33)	
1	38 (16.52)	62 (65.26)		5 (8.77)	18(75.00)		10 (9.26)	11 (55.00)		5 (15.15)	7 (46.67)	
PEF			0.186			0.229			0.986			0.857
0	153 (66.52)	71 (74.74)		33 (57.89)	18(75.00)		83 (76.85)	16 (80.00)		24 (72.73)	12 (80.00)	
1	77 (33.48)	24 (25.26)		24 (42.11)	6(25.00)		25 (23.15)	4 (20.00)		9 (27.27)	3 (20.00)	
Echogenicity			< 0.001			0.004			1.000			0.224
0	168 (73.04)	39 (41.05)		36 (63.16)	6(25.00)		54 (50.00)	10 (50.00)		12 (36.36)	9 (60.00)	
1	62 (26.96)	56 (58.95)		21 (36.84)	18(75.00)		54 (50.00)	10 (50.00)		21 (63.64)	6 (40.00)	
CNA			0.266			1.000			0.675			0.632
0	195 (84.78)	75 (78.95)		44 (77.19)	19(79.17)		90 (83.33)	18 (90.00)		25 (75.76)	13 (86.67)	
1	35 (15.22)	20 (21.05)		13 (22.81)	5(20.83)		18 (16.67)	2 (10.00)		8 (24.24)	2 (13.33)	
Cal			< 0.001			0.002			0.008			0.159
0	219 (95.22)	72 (75.79)		53 (92.98)	15(62.50)		103 (95.37)	15 (75.00)		32 (96.97)	12 (80.00)	
1	11 (4.78)	23 (24.21)		4 (7.02)	9(37.50)		5 (4.63)	5 (25.00)		1 (3.03)	3 (20.00)	
Alder			0.547			0.174			0.207			0.310
0	83 (36.09)	33 (34.74)		26 (45.61)	8(33.33)		20 (18.52)	6 (30.00)		6 (18.18)	6 (40.00)	
I	96 (41.74)	34 (35.79)		13 (22.81)	6(25.00)		28 (25.93)	8 (40.00)		16 (48.48)	5 (33.33)	
II	30 (13.04)	16 (16.84)		6 (10.53)	7(29.17)		24 (22.22)	3 (15.00)		5 (15.15)	3 (20.00)	
III	21 (9.13)	12 (12.63)		12 (21.05)	3(12.50)		36 (33.33)	3 (15.00)		6 (18.18)	1 (6.67)	
LM			< 0.001			< 0.001			0.342			0.011
0	230 (100.00)	74 (77.89)		57 (100.00)	17(70.83)		108 (100.00)	19 (95.00)		33 (100.00)	11 (73.33)	
1	null	21 (22.11)		null	7(29.17)		null	1 (5.00)		null	4 (26.67)	

BPTs, benign parotid gland tumors; MPTs, malignant parotid gland tumor. Gender: 0, female; 1, male. Side: 0, left; 1, right. Shape: 0, regular; 1, irregular. Number: 0, single; 1, multiple. Margin: 0, clear; 1, unclear. PEF (posterior echogenicity feature): 0, no significant change; 1, posterior echogenicity enhancement. Echogenicity: 0, uniform; 1, uneven. Cal (calcification): 0, absence; 1, presence. Alder grading: 0, no flow; I, minimal; II, moderate; III, marked. CNA (cystic or necrotic areas): 0, absence; 1, presence. LM (lymphatic metastasis): 0, absence; 1, presence.

**Table 2:** Comparison of clinical models constructed using machine learning based on features screened by univariate and multivariate analyses of clinical-radiological characteristics.

Model name	Accuracy	AUC	95% CI	Sensitivity	Specificity	PPV	NPV	Cohort
LR	0.831	0.806	0.746 - 0.867	0.926	0.600	0.849	0.770	train
LR	0.877	0.865	0.768 - 0.962	0.947	0.708	0.885	0.850	val
LR	0.859	0.744	0.594 - 0.894	0.935	0.450	0.902	0.562	test1
LR	0.771	0.751	0.584 - 0.917	0.939	0.400	0.775	0.750	test2
Random Forest	0.862	0.918	0.886 - 0.951	0.957	0.632	0.863	0.857	train
Random Forest	0.877	0.914	0.834 - 0.995	0.947	0.708	0.885	0.850	val
Random Forest	0.906	0.889	0.799 - 0.979	0.972	0.550	0.921	0.786	test1
Random Forest	0.667	0.755	0.604 - 0.905	0.788	0.400	0.743	0.462	test2
XGBoost	0.868	0.929	0.900 - 0.958	0.948	0.674	0.876	0.842	train
XGBoost	0.877	0.876	0.774 - 0.978	0.930	0.750	0.898	0.818	val
XGBoost	0.867	0.824	0.723 - 0.924	0.954	0.400	0.896	0.615	test1
XGBoost	0.792	0.790	0.638 - 0.942	0.939	0.467	0.795	0.778	test2

A clinical model was constructed with machine learning based on the selected features. LR, Logistic Regression.

### ROI segmentation evaluation

The performance of models was compared with various evaluation indicators (**Table 3**). Deeplabv3 consistently exhibited excellent performance and outperformed UNet++ and UNet models in terms of multiple indicators, including Dice coefficient, mIOU, FPR, Precision, and Recall (**Supplementary S8**).

**Table 3:** Comparison of the performance of the three automatic segmentation models, Deeplabv3, UNet++, and UNet, in terms of various evaluation indicators.

Model name	Dice	mIOU	FPR	Precision	Recall	Cohort
Deeplabv3	0.971	0.946	0.033	0.969	0.975	train
UNet++	0.954	0.919	0.045	0.958	0.954	train
UNet	0.953	0.918	0.054	0.953	0.957	train
Deeplabv3	0.975	0.952	0.030	0.971	0.979	val
UNet++	0.955	0.921	0.059	0.955	0.961	val
UNet	0.953	0.919	0.075	0.951	0.963	val
Deeplabv3	0.958	0.926	0.056	0.953	0.970	test1
UNet++	0.934	0.889	0.138	0.928	0.954	test1
UNet	0.939	0.896	0.139	0.928	0.963	test1
Deeplabv3	0.960	0.927	0.047	0.955	0.967	test2
UNet++	0.930	0.883	0.073	0.937	0.939	test2
UNet	0.937	0.890	0.073	0.938	0.946	test2

DICE, dice similarity coefficient; IoU, Intersection over Union; FPR, False Positive Rate.

### Visual evaluation results

**Figure 4:**
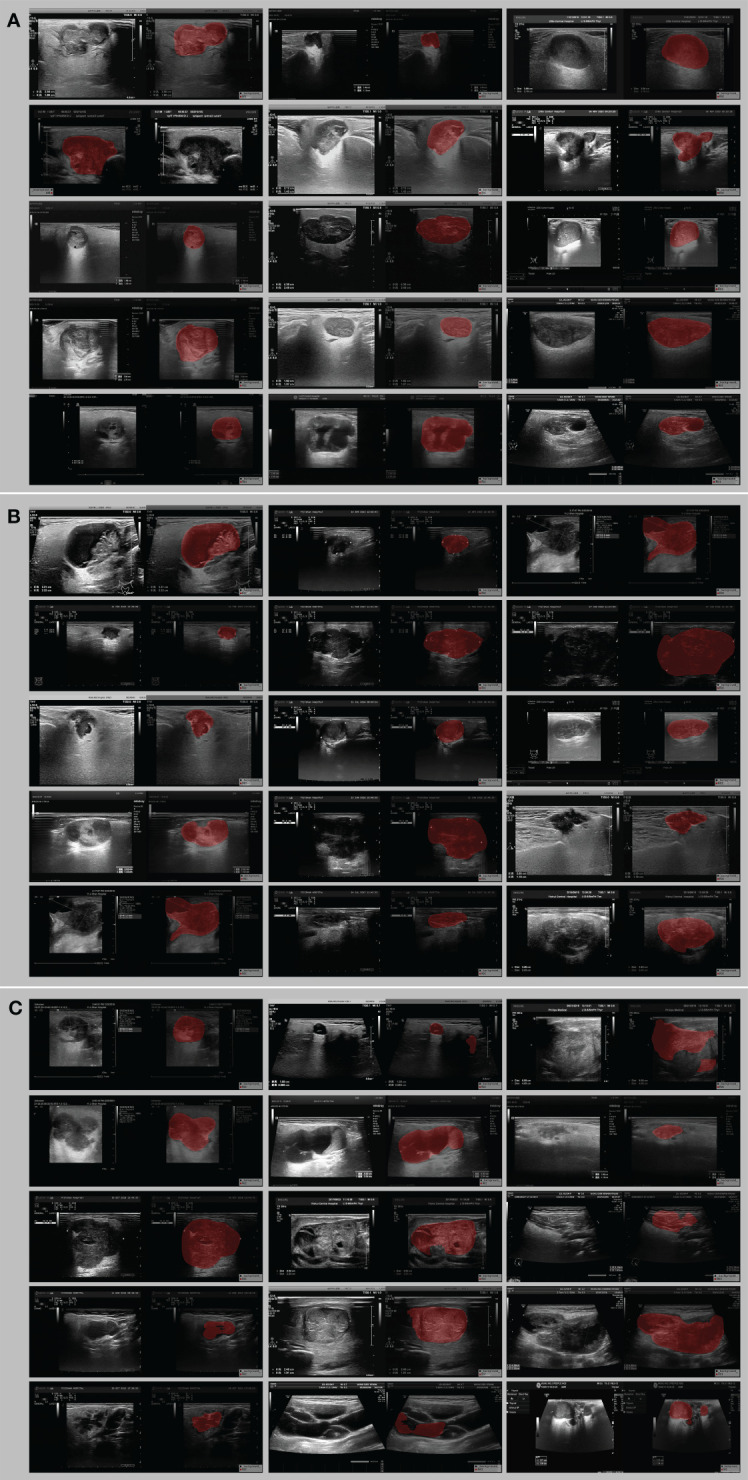
Example image of the best Deeplabv3 segmentation model for lesion delineation on ultrasound images. **(A)** all three physicians were satisfied with the visual assessment, **(B)** one of the three physicians was dissatisfied with the segmentation, and **(C)** two or three of the three physicians were dissatisfied with the segmentation.

Based on the quantization results, the optimal segmentation model (DeeplabV3) was selected. The outputs of the three models were visualized as models on the dataset to visually analyze differences in segmentation results of our proposed automatic segmentation model and the other 2 models. **Table 4** depicts the results of the qualitative comparison. As observed, the segmentation results of the proposed Deeplabv3+ model were superior to those of U-Net and U-Net++, which had some subtle visible errors, The Deeplabv3+ model exhibited higher reproducibility than the other 2 models. To further increase the reliability of clinical trials, the Deeplabv3+ prediction results were presented to three experienced experts who were invited to evaluate our segmentation results through majority voting. Approximately 96% of the predicted segmentation results were endorsed by the experts, highlighting the effectiveness of our approach in a real-world clinical setting (**Figure 4**).

**Table 4:** Comparison of performance of six different DL models.

Model name	Acc	AUC	95%CI	Sensitivity	Specificity	PPV	NPV	Cohort
Densenet201	0.794	0.822	0.7718-0.8731	0.495	0.917	0.712	0.815	train
Densenet201	0.691	0.780	0.6735-0.8857	0.125	0.930	0.429	0.716	val
Densenet201	0.836	0.759	0.6466-0.8719	0.200	0.954	0.444	0.866	test1
Densenet201	0.625	0.733	0.5862-0.8804	0.067	0.879	0.200	0.674	test2
Inception_v3	0.732	0.703	0.6388-0.7671	0.316	0.904	0.577	0.762	train
Inception_v3	0.765	0.702	0.5626-0.8409	0.500	0.877	0.632	0.806	val
Inception_v3	0.648	0.670	0.5316-0.8092	0.600	0.657	0.245	0.899	test1
Inception_v3	0.688	0.754	0.5958-0.9113	0.733	0.667	0.500	0.846	test2
ResNet101	0.782	0.803	0.7505-0.8551	0.389	0.943	0.740	0.789	train
ResNet101	0.704	0.705	0.5728-0.8373	0.542	0.772	0.500	0.800	val
ResNet101	0.594	0.727	0.6144-0.8393	0.650	0.583	0.224	0.900	test1
ResNet101	0.771	0.824	0.7059-0.9426	0.867	0.727	0.591	0.923	test2
ResNet18	0.772	0.811	0.7613-0.8615	0.453	0.904	0.662	0.800	train
ResNet18	0.765	0.808	0.6942-0.9228	0.750	0.772	0.581	0.880	val
ResNet18	0.711	0.809	0.7119-0.9062	0.600	0.731	0.293	0.908	test1
ResNet18	0.667	0.812	0.6802-0.9441·	0.733	0.636	0.478	0.840	test2
ResNet50	0.812	0.852	0.8085-0.8959	0.537	0.926	0.750	0.829	train
ResNet50	0.704	0.788	0.6782-0.8986	0.542	0.772	0.500	0.800	val
ResNet50	0.711	0.683	0.5493-0.8173	0.550	0.741	0.282	0.899	test1
ResNet50	0.667	0.735	0.5951-0.8756	0.600	0.697	0.474	0.793	test2
VGG19	0.766	0.755	0.6963-0.8128	0.379	0.926	0.679	0.783	train
VGG19	0.778	0.763	0.6433-0.8830	0.333	0.965	0.800	0.775	val
VGG19	0.742	0.700	0.6013-0.7992	0.100	0.861	0.118	0.838	test1
VGG19	0.833	0.812	0.6812-0.9430	0.667	0.909	0.769	0.857	test2

AUC, area under the receiver-operating characteristic curve; PPV, positive predictive value; NPV, negative predictive value.

The above quantification and visual evaluation results illustrated that the Deeplabv3 model had the best segmentation performance, thus laying the foundation for subsequent model construction.

### DL signature

**Figure 5:**
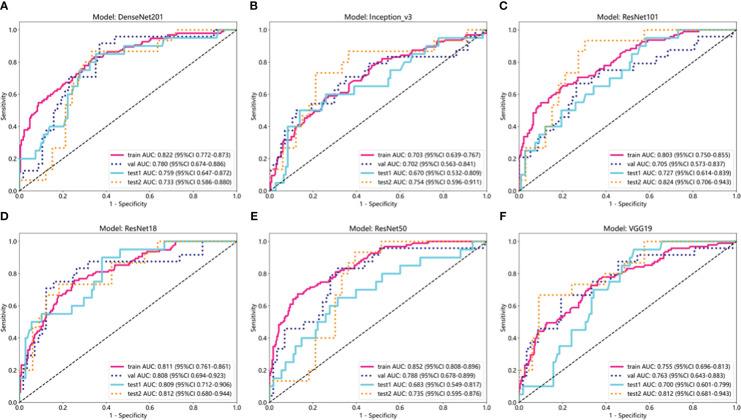
ROC curves of six deep learning models in internal training and validation sets and external test sets 1 and 2. **(A)** ROC curve of DenseNet201; **(B)** ROC curve of Inception_v3; **(C)** ROC curve of ResNet101; **(D)** ROC curve of ResNet18; **(E)** ROC curve of ResNet50; **(F)** ROC curve of VGG19.

The results of the DL models (**Table 4**) demonstrated that the ResNet18 model outperformed other models in terms of classification performance. ResNet18 exhibited a minimal loss value during training (22), indicating fewer errors, and achieved a higher convergence rate compared to other CNNs (**Supplementary Figure 1**). In the internal validation set, ResNet18 achieved an AUC of 0.808 (95%CI: 0.694−0.923), an accuracy of 0.765, a sensitivity of 0.750, and a specificity of 0.772. Additionally, in external test sets 1 and 2, ResNet18 maintained favorable classification performance with AUCs of 0.809 (95%CI: 0.712−0.906) and 0.812 (95%CI: 0.680-0.944), accuracies of 0.711 and 0.667, sensitivities of 0.600 and 0.733, and specificities of 0.731 and 0.636, respectively. Although the ResNet18 model did not surpass the clinical model in overall performance, it showed comparable AUCs in all test sets. Specifically, the AUCs of ResNet18 and the clinical models were 0.808 and 0.876 (*p* = 0.264) in the internal validation set, 0.809 and 0.824 (*p* = 0.814) in external test set 1, and 0.809 and 0.824 (*p* = 0.809) in external test set 2 (**Figure 5**).

**Figure 6:**
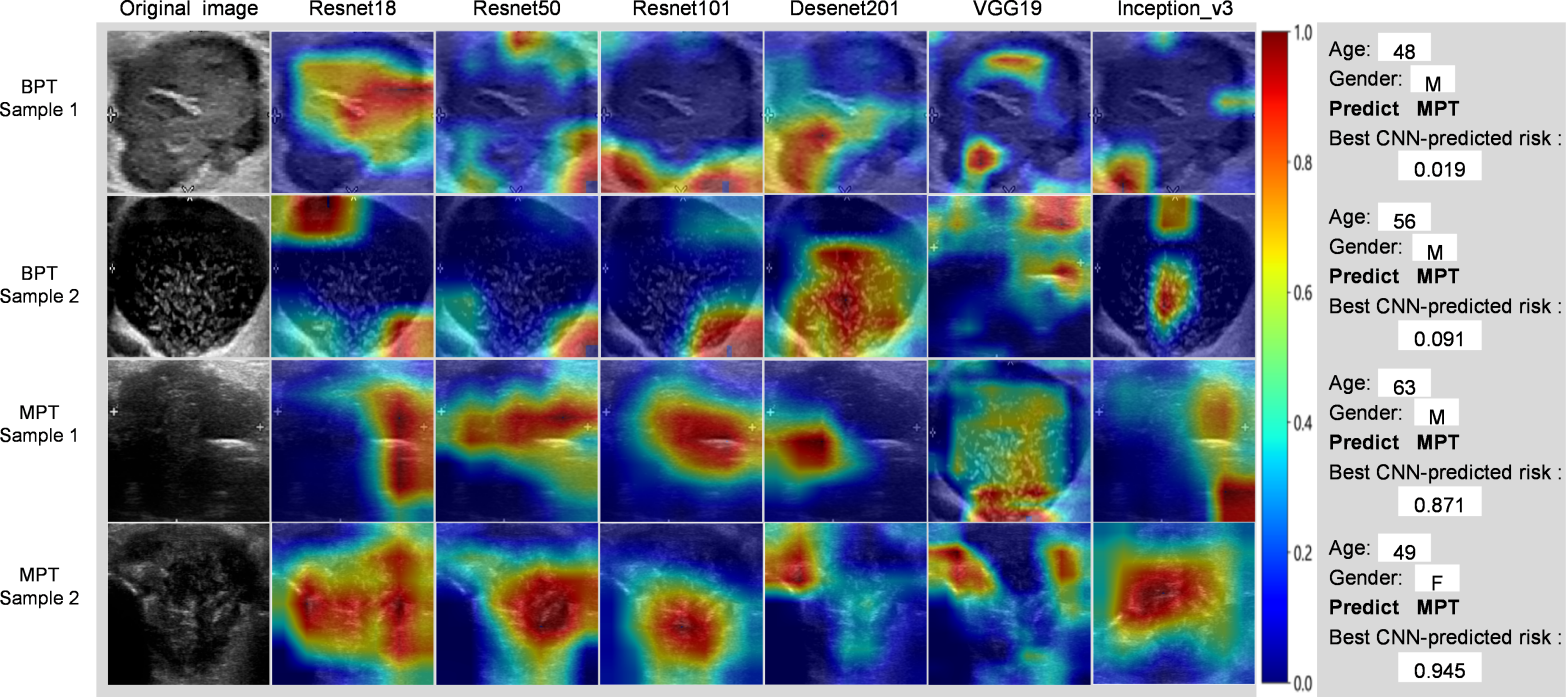
Grad-CAM visualization of 4 typical samples. The attention regions of diferent deep learning models in benign and malignant parotid tumors US image analysis; BPT, benign parotid gland tumor; MPT, malignant parotid gland tumor.

In terms of model interpretability, ResNet18 exhibited clearer regions of interest (ROIs), focusing on the margin and internal regions of the tumor, while not activating regions adjacent to normal parotid tissues (**Figure 6**).

### Diagnostic performance of the radiologist and DL model–assisted diagnosis

The diagnostic performance of radiologists in distinguishing BPTs from MPTs under the aid of ResNet18 is summarized in **Table 5**. The overall performance of radiologists, including those with different levels of experience, was significantly enhanced with the assistance of the ResNet18 model. For radiologist A, the AUC increased by 0.082 (*p* = 0.012) in the internal validation set, 0.084 (*p* = 0.042) in external test set 1, and 0.064 (*p* = 0.107) in external test set 2. For radiologist B, the AUC improvement was 0.129 (*p* = 0.001) in the internal validation set, 0.092 (*p* = 0.016) in external test set 1, and 0.149 (*p* = 0.022) in external test set 2. Radiologist C saw AUC enhancements of 0.106 (*p* = 0.014), 0.080 (*p* = 0.007), and 0.149 (*p* = 0.030) in the internal validation set, external test set 1, and external test set 2, respectively.

**Table 5:** Performance comparison between ResNet18 and radiologists of different seniorities and between radiologists with and without model assistance.

	Accuracy	AUC	95%CI	Sensitivity	Specificity	PPV	NPV	Cohort
Clinical	0.889	0.886	0.7842 - 0.9870	0.750	0.947	0.857	0.900	val
Deep Learning	0.765	0.808	0.6942 - 0.9228	0.750	0.772	0.581	0.880	val
Radiologist-A	0.778	0.770	0.6664 - 0.8731	0.750	0.789	0.600	0.882	val
Radiologist-B	0.704	0.705	0.5945 - 0.8156	0.708	0.702	0.500	0.851	val
Radiologist-C	0.716	0.702	0.5895 - 0.8140	0.667	0.737	0.516	0.840	val
DLR-assisted RA	0.877	0.852	0.7611 - 0.9429	0.792	0.912	0.792	0.912	val
DLR-assisted RB	0.852	0.834	0.7410 - 0.9279	0.792	0.877	0.731	0.909	val
DLR-assisted RC	0.815	0.808	0.7113 - 0.9049	0.792	0.825	0.655	0.904	val
Clinical	0.867	0.824	0.7231 - 0.9241	0.400	0.954	0.615	0.896	test1
Deep Learning	0.711	0.809	0.7119 - 0.9062	0.600	0.731	0.293	0.908	test1
Radiologist-A	0.891	0.793	0.6829 - 0.9023	0.650	0.935	0.650	0.935	test1
Radiologist-B	0.797	0.737	0.6239 - 0.8502	0.650	0.824	0.406	0.927	test1
Radiologist-C	0.773	0.703	0.5864 - 0.8191	0.600	0.806	0.364	0.916	test1
DLR-assisted RA	0.930	0.877	0.7847 - 0.9690	0.800	0.954	0.762	0.963	test1
DLR-assisted RB	0.883	0.829	0.7276 - 0.9299	0.750	0.907	0.600	0.951	test1
DLR-assisted RC	0.875	0.783	0.6729 - 0.8937	0.650	0.917	0.591	0.934	test1
Clinical	0.792	0.790	0.6382 - 0.9416	0.467	0.939	0.778	0.795	test2
Deep Learning	0.667	0.812	0.6802 - 0.9441	0.733	0.636	0.478	0.840	test2
Radiologist-A	0.833	0.806	0.6772 - 0.9349	0.733	0.879	0.733	0.879	test2
Radiologist-B	0.792	0.739	0.5992 - 0.8796	0.600	0.879	0.692	0.829	test2
Radiologist-C	0.750	0.709	0.5644 - 0.8538	0.600	0.818	0.600	0.818	test2
DLR-assisted RA	0.896	0.870	0.7571 - 0.9823	0.800	0.939	0.857	0.912	test2
DLR-assisted RB	0.896	0.888	0.7859 - 0.9899	0.867	0.909	0.812	0.937	test2
DLR-assisted RC	0.854	0.858	0.7490 - 0.9661	0.867	0.848	0.722	0.933	test2

AUC, area under the receiver-operating characteristic curve; PPV, positive predictive value; NPV, negative predictive value; Radiologist A represents senior physicians, radiologist B represents attending physicians, and radiologist C represents resident physicians. DLR-assisted RA, deep learning radiomics-assisted radiologist A; DLR-assisted RB, deep learning radiomics-assisted radiologist B; DLR-assisted RC, deep learning radiomics-assisted radiologist C.

**Figure 7:**
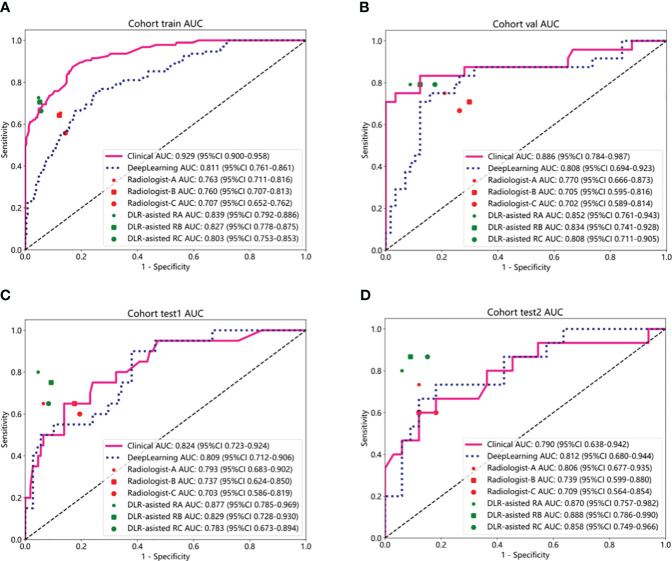
Performance comparison between clinical model and deep learning model (ResNet18). **(A-D)** Communication between radiologists with and without ResNet18 assistance to discriminate between BPTs and MPTs in the internal train set **(A)**, the internal validation set **(B)**, external test set 1 **(C)**, and external test set 2 **(D)**. Radiologist A represents senior physicians, radiologist B represents attending physicians, and radiologist C represents resident physicians. DLR-assisted RA, deep learning radiomics-assisted radiologist A; DLR-assisted RB, deep learning radiomics-assisted radiologist B; DLR-assisted RC, deep learning radiomics-assisted radiologist C.

The diagnostic performance of the ResNet18 model was comparable to that of senior radiologist A, and significantly better than radiologists B and C (**Figure 7**). The kappa values for radiologists B and C improved from 0.615 to 0.808 in the internal validation set, from 0.814 to 0.870 in external test set 1, and from 0.698 to 0.727 in external test set 2 with the assistance of ResNet18. The clinical diagnostic benefit of radiologists aided by the ResNet18 DL model was superior to that of independent visual assessments by radiologists. In the internal validation set, the NRI values for radiologists A, B, and C were 0.164 (*p* = 0.015), 0.259 (*p* = 0.002), and 0.213 (*p* = 0.017), respectively, with corresponding IDI values of 0.235 (*p* = 0.016), 0.284 (*p* = 0.005), and 0.201 (*p* = 0.043). In external test set 1, the NRI values for radiologists A, B, and C were 0.169 (*p* = 0.054), 0.183 (*p* = 0.019), and 0.161 (*p* = 0.008), respectively, with IDI values of 0.204 (*p* = 0.068), 0.205 (*p* = 0.031), and 0.184 (*p* = 0.045). In external test set 2, the NRI values for radiologists A, B, and C were 0.127 (*p* = 0.109), 0.297 (*p* = 0.038), and 0.297 (*p* = 0.047), with IDI values of 0.194 (*p* = 0.132), 0.332 (*p* = 0.031), and 0.294 (*p* = 0.046), respectively.

## Discussion

Although the preferred treatment for PGTs is surgery, various histological types of PGTs lead to obvious differences in treatment decisions and prognosis (23). Therefore, accurate differentiation of BPTs from MPTs is the key to clinical treatment decisions and prognosis of patients. Identification and segmentation of tumor regions are a prerequisite for tumor evaluation, which also simplifies subsequent clinical evaluation. In clinical practice, PTG segmentation is, however, usually performed by clinicians manually with semi-automatic methods and is followed by pixel layering, which is time-consuming and laborious. As medical image data grow dramatically, there is an increasingly urgent clinic for automatic segmentation methods of medical images.

In recent years, artificial intelligence (AI) has been extensively applied to medical image segmentation. Nevertheless, no research pertaining to PGT segmentation has been published. As such, our study evaluated the feasibility of DL for PGT segmentation. Ultrasound images of 582 patients with PGTs from four hospitals were included in the present study. Five evaluation indicators were utilized to analyze the segmentation performance of different models. The Deeplabv3+ model yielded the optimal segmentation results in both internal and external test sets, with a Dice similarity coefficient of 0.975, 0.958, and 0.960 in the internal validation set and external test sets 1 and 2, respectively, implicating higher accuracy and generalization of the Deeplabv3 + model. This study majorly aimed to construct a model for PGT segmentation based on ultrasound images and further develop a model incorporating the optimal automatic segmentation model and DL methods, thus increasing workflow efficiency and assisting radiologists in diagnosis. Our results elucidated that despite the presence of several subtle visible errors in the segmentation results of all U-Net, Deeplabv3+, and U-Net++, the segmentation results of the Deeplabv3+ model were more factual in terms of both contour and detail. In addition, the repeatability of the Deeplabv3+ model was higher than that of the other two models, as qualitatively demonstrated by the quantitative values of Dice, mIOU, FPR, and Precision in the internal validation set and external test sets 1 and 2.

At present, surgery is the predominant treatment strategy for PGTs, and the preoperative differential diagnosis of BPTs and MPTs is essential for surgical planning (9). As reported, BGTs can be treated with local excision or lateral parotidectomy. While the treatment of MPTs generally involves radical parotidectomy, such as extended resection combined with lymph node dissection, or even facial nerve resection and postoperative radiotherapy, which may evoke more complications and invasive injuries for patients (12, 23). Although USCB and FNA can differentiate BPTs from MPTs to some extent, both methods are accompanied by serious complications since they are invasive. Due to the overlapping imaging features of different types of PGTs, traditional visual assessment is commonly affected by confounding factors. Of note, PGTs are usually located in the superficial lobe and are readily imaged by routine ultrasound, thus predisposing ultrasound to be an ideal examination method for PGTs. Ultrasound is a broadly accepted technique for PGT screening and is applied to diagnose most PGTs because it is sensitive, non-invasive, quick, radiation-free, and inexpensive. Nonetheless, the accurate diagnosis of PGTs by ultrasound is largely determined by the professional experience and expertise of radiologists. A former study reported that although the sensitivity and specificity of B-mode ultrasound were 38.9% and 90.1% in differentiating malignant and benign parotid nodules, respectively, its accuracy in differentiating malignant masses was only 20% (13). Consequently, more reliable methods to distinguish PGTs are required to be developed, thereby improving the differential diagnosis of PGTs by ultrasound. In this context, this study explored whether satisfactory results could be acquired by using an automated segmentation model under the aid of the optimal DL model, without the use of invasive methods. ML has been confirmed to be more advantageous in classification tasks than visual assessment by a radiologist since it can first learn features from medical images to maintain consistency and repeatability in diagnosis.

The present study assessed the value of several DL systems with different deep neural networks in the identification of PGTs. DL has achieved end-to-end classification and prediction. Many published articles have reported that DL can be used for the diagnosis and management of various tumors. Additionally, there are also multiple studies on the use of DL to characterize PGTs. For example, Wang et al. (12) reported that four DL methods could distinguish BPTs and MPTs based on ultrasound images with an AUC of 0.80−0.82. Xia et al. (24) developed a DL algorithm with an accuracy of 82.18% (95%CI = 0.77, 0.86) in the diagnosis and staging of parotid gland cancer based on MRI images. Some studies (18, 25) constructed DL-assisted diagnostic models based on CT images to improve the differential diagnosis of BPTs and MPTs by radiologists. At present, DL based on ultrasound images is yet not applied to the identification of the nature of PGTs, particularly to automatic segmentation of images. The study incorporated a relatively large sample size and two independent external test sets. Ultrasound images were analyzed to train an optimal segmentation model, which was subsequently used to develop a deep learning (DL) model aimed at assisting radiologists in diagnosis. Although the robust performance of these models may be attributed to radiologists, aided by the DL models, having reviewed the cases and gained certain insights for subsequent diagnoses, the study design introduces uncertainty regarding whether the enhanced accuracy of the radiologists was due to repeated case reviews or the assistance of the DL model. This ambiguity constitutes a major limitation of our study. Nonetheless, the study demonstrated a positive impact, indicating that our findings possess significant clinical value.

In this study, six DL models were utilized to distinguish BPTs from MPTs. ResNet18 has more robust generalization and general prediction capabilities when compared with other classical CNNs, which may be associated with different performance of diverse CNN models due to differences in the internal structure of the network. ResNet can train very deep neural networks to prevent the gradient disappearance problem and improve the expression ability and performance of the model (26). The use of residual connections can preserve the original features, which smoothes and stabilizes the learning of the network and further elevates the accuracy and generalization ability of the model. ResNet has the advantages of detailed and accurate description and strong robustness and is more suitable for training on small datasets. ResNet is a deep residual network proposed by He et al (26). in 2016 to address the degradation issue in deep neural networks, in which the network structure is connected by introducing residuals (residual connections) to overcome the problems of gradient disappearance and explosion, which enables the training of deeper networks. Several studies (27–29) have demonstrated that ResNet18 is well implemented for image classification and has been applied to medical image research. In our study, the Grad-CAM visualization of ResNet18 exhibited two key regions of tumors (margin and inner region), facilitating the distinguishment of BPTs from MPTs. Compared with BPTs, MPTs have a more irregular shape, more blurred margin, and more complex internal tumor heterogeneity. Therefore, these two key regions correspond to the areas on which radiologists focus, which supports the validity of the model to a certain extent. In addition, with the aid of the optimal model, the clinical benefit of radiologists with different seniorities was improved to some extent. In the internal validation set, NRI values of radiologists B and C were 0.259 (*p* = 0.002) and 0.213 (*p* = 0.017), and their IDI values were 0.284 (*p* = 0.005) and 0.201 (*p* = 0.043), respectively. In external test set 1, NRI values of radiologists B and C were 0.183 (*p* = 0.019) and 0.161 (*p* = 0.008), with IDI values of 0.205 (*p* = 0.031) and 0.184 (*p* = 0.045), respectively. In external test set 2, radiologists B and C showed NRI values of 0.297 (*p* = 0.038) and 0.297 (*p* = 0.047) and IDI values of 0.332 (*p* = 0.031) and 0.294 (*p* = 0.046), respectively. In addition, the diagnostic performance of junior and attending radiologists was also enhanced, indicating the developed DL model based on the automatic segmentation model as a potential aid to quickly assist radiologists in optimizing radiological interpretation and reducing misdiagnosis due to inexperience.

This study has several limitations. First, this study is a retrospective study, which calls for the validation of our results in prospective clinical trials before the assistive system can be applied in the clinic. Second, few pathological types of MPTs existed in external test sets 1 and 2. Third, this study only focused on the differential diagnosis of BPT and MPT without further investigating pathological classification. Fourth, the developed optimal automatic segmentation model may result in some result bias due to no manual adjustment. Accordingly, further studies are warranted to investigate whether the combination of automatic segmentation model training with manual tuning can further improve the performance of this model. Fifth, patients underwent ultrasound on non-uniform instruments in different centers. Sixth, the main limitation of the study is likely the bias due to the radiologists having already viewed the cases when they were being assisted by the deep learning model. The way the study is designed makes it uncertain whether the increased accuracy of the radiologists is due to the repeat reviewing of the cases or due to the assistance from the model. Lastly, Developing ML models that incorporate a wider range of radiological and multimodal images is the future of medicine; however, due to the retrospective nature of our study, a combined model based on Color Doppler Flow Imaging(CDFI), elastography, and contrast-enhanced ultrasound images(CEUS) for patients with PGT has not been established, making this an important direction for our future research.

## Conclusion

In this study, the automatic segmentation model can effectively segment parotid lesions on ultrasound images to a certain extent. On the basis of the segmentation model, we proposed a DL-aided diagnostic model, which displayed excellent performance in differentiating BPTs from MPTs and compensated the gap between the experience levels of different radiologists to foster the precise treatment of patients with PGTs.

## Data Availability

The raw data supporting the conclusions of this article will be made available by the authors, without undue reservation.
