# Supplementary material for: Deep learning-assisted diagnosis of benign and malignant parotid gland tumors based on automatic segmentation of ultrasound images: a multicenter retrospective study
Source: Front Oncol. 2024 Aug 9;14:1417330. doi: 10.3389/fonc.2024.1417330 (PMC11341398; doi:10.3389/fonc.2024.1417330)
Supplement: Supplementary file 1 [file DataSheet_1.docx]

**Supplementary Material**

**1 Histopathological types and numbers**

**Table S1** shows the histopathological types and numbers of all patients with parotid tumors admitted to the four centers.

|  | Internal training and validation set | External validation set 1 | | External validation set 1 |
| --- | --- | --- | --- | --- |
|  | Center1 | Center2 | Center3 | Center4 |
| Pleomorphic adenoma | 159 | 38 | 11 | 15 |
| Warthin tumor | 83 | 31 | 10 | 10 |
| Basal cell adenoma | 25 | 6 | 0 | 4 |
| Others BPTs | 20 | 12 | 0 | 4 |
| Mucoepidermoid carcinoma | 21 | 3 | 3 | 3 |
| Adenoid cystic carcinoma | 20 | 0 | 1 | 4 |
| Acinic cell carcinoma | 18 | 1 | 2 | 0 |
| Adenocarcinoma | 0 | 1 | 0 | 1 |
| Squamous cell carcinoma | 18 | 0 | 2 | 5 |
| Basal cell adenocarcinoma | 2 | 0 | 0 | 0 |
| Salivary ductal carcinoma | 12 | 0 | 1 | 0 |
| Myoepithelial carcinoma | 13 | 1 | 0 | 0 |
| Lymphoepithelial carcinoma | 12 | 1 | 0 | 0 |
| Others MPTs | 3 | 1 | 3 | 2 |

BPTs, benign parotid gland tumors; MPTs, malignant parotid gland tumors.Center 1, The First Affiliated Hospital of Wannan Medical College (Yijishan Hospital); External validation set 1; Center 2, WuHu Hospital, East China Normal University (The Second People’s Hospital, WuHu) and center 3, Zibo Central Hospital; External validation set 2, Linyi Central Hospital.

**2 US protocol:**

All patients at four centers underwent US within 1 month before surgery. Table S2 lists the four central US device probes and frequency ranges.

**Table S2.** US protocols of the four centers

|  | US Device Name | Place of production | Probe | Range of Frequencies |
| --- | --- | --- | --- | --- |
| Center1 | Esaote Mylab Twice | Genova, ITALY | LA523 | 4-13 MHz |
|  | Siemens S2000 | CA, USA | L4-9 | 4-9 MHz |
|  | PHILIPS EPIQ 5 | CA, USA | eL18-4 | 4-18MHz |
| Center2 | Siemens ACUSON Sequoia | CA, USA | 10L4 | 4-10MHz |
|  | PHILIPS EPIQ7 | CA, USA | eL18-4 | 4-18MHz |
| Center3 | HITACHI ALOKA | ToKyo,Japan | L55 | 5-13 MHz |
|  | PHILIPS EPIQ 5 | CA, USA | L12-5 | 6-15 MHz |
|  | Esaote Mylab Twice | Genova, ITALY | LA523 | 4-13 MHz |
|  | GE E9 | ToKyo,Japan | 9L | 6-15 MHz |
| Center4 | PHILIPS EPIQ7C | CA, USA | L12-5 | 6-15 MHz |
|  | Mindray Resona 7EXP | Shenzhen，China | L14-5WU | 7-14MHz |

**S3. Loss Function**:

Loss Function: We used DiceCELoss, which combines the Dice Loss and Cross-Entropy Loss functions:

$$Loss=-\frac{1}{N}\sum_{i=1}^{N} \left( \alpha\log\left( \frac{2}{\frac{1}{\epsilon+Dice_{i}}+\frac{1}{\epsilon+CE_{i}}} \right)+\left( 1-\alpha\right)\log\left( \epsilon+Dice_{i} \right) \right)$$

Here, $N$ is the number of samples in a batch, $Dice_{i}$ and $CE_{i}$ are the values of Dice Loss and Cross-Entropy Loss functions for the $i^{th}$ sample, $\alpha$ is a weight parameter used to balance the contribution of the two loss functions. Setting the weights of unlabeled pixels to zero makes it possible to learn from only the labelled ones and, hence, to generalize to the whole volume.

**S4. Our learning rate is presented as follows:**

$$\eta_{t}=\eta_{min}^{i}+\frac{1}{2}\left( \eta_{max}^{i}-\eta_{min}^{i} \right)\left( 1+cos\left( \frac{T_{cur}}{T_{i}}\pi\right) \right)$$

**S5. Statistical analysis**

The data were analyzed using Python software (version 3.9.16) , R software (4.1.0)and SPSS software (version 27.0, IBM Corporation, Armonk, New York). Kolmogorov-Smirnow test and Levene test were used to test the normality of continuous quantitative data. The data in accordance with normal distribution were expressed as mean standard deviation (xˉ±s) and analyzed by student t test. The non-normal distribution data were expressed as median and interquartile range M (Q1, Q3), and the non-parametric Mann-Whitney U test was used. Continuous data were compared student t test was used for comparison. Categorical data were presented as numbers and percentages using categorical variables. Chi-square test or Fisher exact test was used for statistical analysis.

Multiple logistic regression analysis with forward stepwise selection was used to identify independent signatures predicting MPTs in the clinical dataset. To account for the potential effect of clinical features on each patient, we constructed clinical models（Clinical） with multiple machine learning approaches. With the assistance of the model, Net reclassification index (NRI) and Integrated discrimination Improvement (IDI) were used to evaluate clinical benefit. We generated ROC curves to evaluate the diagnostic performance of the models. DeLong or Hanley & McNeil test used to assess the difference between each model of the AUC.A two-sided *P* <0.05 was used to indicate a statistically significant

**6. Table S3. Consistency test of radiologist A and B in the evaluation of US morphological features of PGTs.**

| **US morphological features** |  | **Kappa value** |  | **P-value** |
| --- | --- | --- | --- | --- |
| Side |  | 1.000 |  | ＜0.001 |
| Number |  | 1.000 |  | ＜0.001 |
| Shape |  | 0.892 |  | ＜0.001 |
| Boundary |  | 0.885 |  | ＜0.001 |
| PEF |  | 0.930 |  | ＜0.001 |
| CNA |  | 0.951 |  | ＜0.001 |
| Cal |  | 0.890 |  | ＜0.001 |
| Echogenicity |  | 0.944 |  | ＜0.001 |
| Alder |  | 0.977 |  | ＜0.001 |
| LM |  | 0.984 |  | ＜0.001 |

PEF, posterior echo feature , CAl, calcification ,CNA, cystic or necrotic areas; LM,lymphatic metastasis

**7. Assessment of clinical-US imaging features**

We conducted univariate analysis on all clinical features, calculating the Odds ratio (OR) and corresponding p-values for each variable. The clinical-US imaging features with statistical significance in all cohorts were screened by univariate analysis, including Age, Echogenicity, Shape, Cal and LM. Then, multivariate logistics regression analysis was used to screen out the independent risk factors for predicting parotid gland malignant tumors, and a clinical diagnostic model was constructed. Finally, we found that shape, boundary, and LM were independent risk factors for US diagnosis of MPTs. Therefore, they were incorporated into the construction of clinical models.

**Table S4,Univariable and Multivariable Analysis of clinical features.**

| feature_name | OR | OR lower 95%CI | OR upper 95%CI | p_value | OR | OR lower 95%CI | OR upper 95%CI | p_value |
| --- | --- | --- | --- | --- | --- | --- | --- | --- |
| Age | 1.004 | 1.001 | 1.006 | 0.010 | 1.001 | 0.999 | 1.003 | 0.506 |
| Gender | 0.992 | 0.919 | 1.069 | 0.856 |  |  |  |  |
| Size | 1.001 | 0.998 | 1.005 | 0.520 |  |  |  |  |
| Side | 0.969 | 0.887 | 1.059 | 0.559 |  |  |  |  |
| Number | 0.916 | 0.799 | 1.049 | 0.287 |  |  |  |  |
| PEF | 0.910 | 0.840 | 0.985 | 0.050 |  |  |  |  |
| Alder | 1.027 | 0.989 | 1.066 | 0.249 |  |  |  |  |
| CNA | 1.062 | 0.964 | 1.170 | 0.307 |  |  |  |  |
| Echogenicity | 1.337 | 1.244 | 1.438 | 0.000 | 1.035 | 0.961 | 1.115 | 0.451 |
| Shape | 1.406 | 1.313 | 1.507 | 0.000 | 1.114 | 1.035 | 1.200 | 0.017 |
| Cal | 1.550 | 1.387 | 1.733 | 0.000 | 1.065 | 0.951 | 1.192 | 0.360 |
| Boundary | 1.670 | 1.557 | 1.790 | 0.000 | 1.413 | 1.294 | 1.542 | <0.001 |
| LM | 1.906 | 1.679 | 2.164 | 0.000 | 1.469 | 1.296 | 1.667 | <0.001 |

PEF, posterior echo feature; CNA, cystic or necrotic areas; Cal, calcification;LM,lymphatic metastasis

S8. ROI **Segmentation Evaluation**

In the segmentation process, we used the Dice similarity coefficient (DICE) for evaluation. The DICE coefficient is a measure of similarity that is often used to quantify the overlap between two samples, and is calculated as:


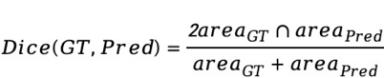


In addition to DICE, we also used other evaluation metrics, including the Intersection over Union (IoU), False Positive Rate(FPR), Precision and Recall. The formulas for these metrics are:


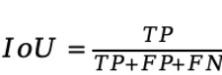
,
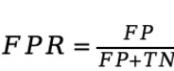
,
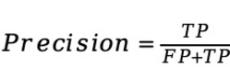
,
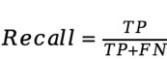


where TP, FP, and FN are the number of true positive, false positive, and false negative.


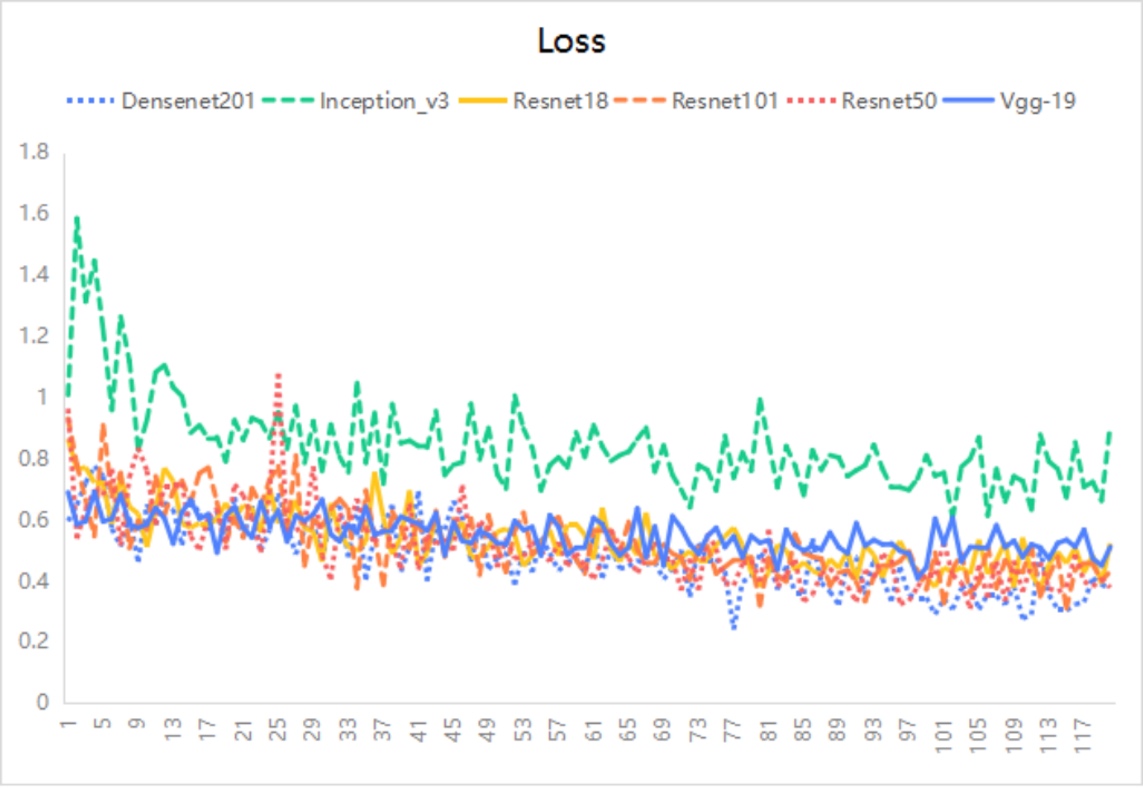


Fig 1 The loss values of six different DL models in the training set according to iteration steps
